# Coronavirus Disease 2019-Associated Bilateral Massive Pulmonary Emboli Caused Death in a Healthy 35-Year-Old Patient

**DOI:** 10.7759/cureus.10213

**Published:** 2020-09-02

**Authors:** Ali F Al Sbihi, Nouraldeen Manasrah, Jorgena Kosti, Sulaiman Alhassan

**Affiliations:** 1 Internal Medicine, Detroit Medical Center Sinai-Grace Hospital, Detroit, USA; 2 Internal Medicine, Hematology, Oncology, Ascension Providence Hospital, Detroit, USA; 3 Internal Medicine, Intensive Care, Pulmonary Medicine, Detroit Medical Center Sinai-Grace Hospital, Detroit, USA

**Keywords:** coronavirus disease (covid-19), massive pulmonary embolism, covid-19 infection

## Abstract

Coronavirus disease 2019 (COVID-19) infection was first reported in December 2019. Within three months, the virus caused a global pandemic that has affected the whole world’s dynamics. Many causes of death due to COVID-19 infection have been identified, involving but not limited to atypical acute respiratory distress syndrome, hypercoagulability, renal failure, and a proinflammatory cytokine storm, often associated with multiorgan failure. We report the case of a young, previously healthy patient who developed massive pulmonary emboli due to COVID-19 infection, resulting in death.

## Introduction

The outbreak of COVID-19 in an unprecedented world event that has affected millions. The disease is associated with coagulopathy, often with thrombotic complications seen in the critically ill patients, which can worsen their severity and in many cases lead to death. Recently, cases of acute pulmonary embolism (PE) associated with severe COVID-19 infection, more commonly present in the elderly, have been reported and discussed [1]. Here we report a case of COVID-19, in an unusual category of patients, with a very rapid and devastating outcome.

## Case presentation

**Figure 1:**
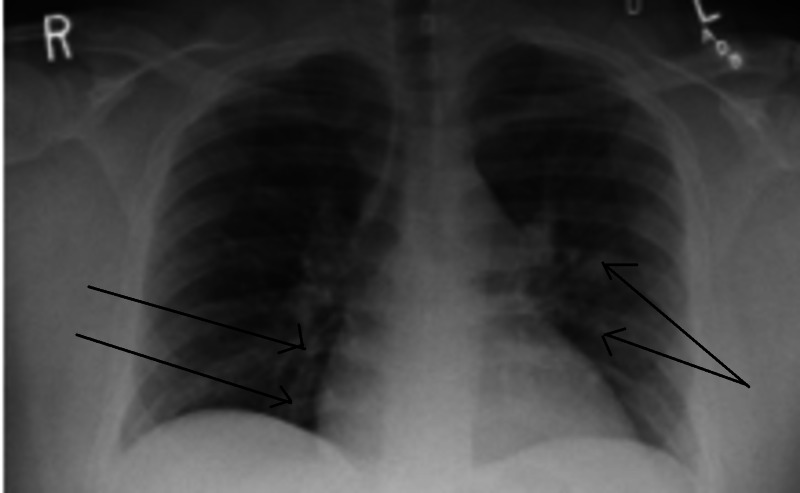
Chest X-ray Patchy opacities in bilateral lung fields

**Figure 2:**
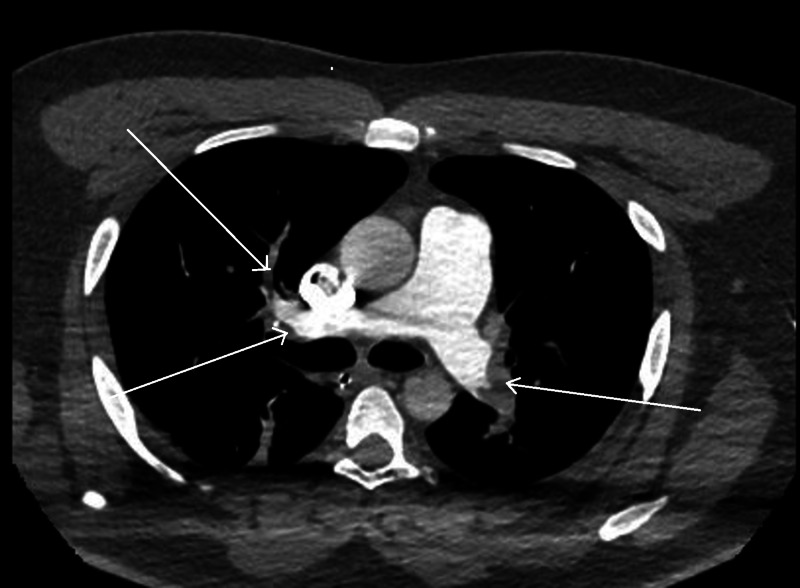
Computed Tomography –Angiography (CT-PA) of the chest, mediastinal window Large filling defects in the main, segmental, and subsegmental branches of pulmonary arteries

**Figure 3:**
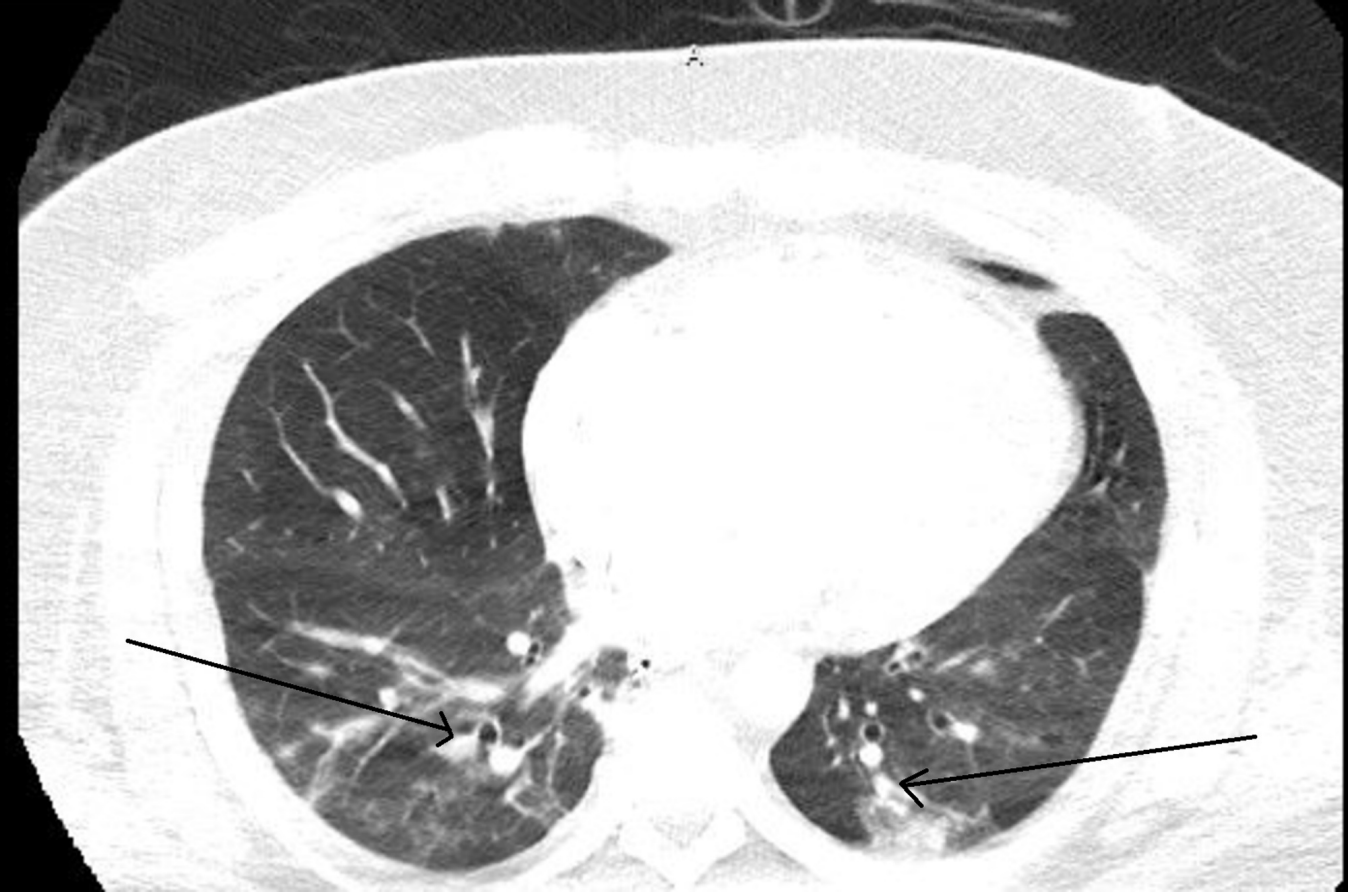
CT-Pulmonary angiogram of the chest, lung window Bilateral ground glass opacities are noted in the lungs bilaterally in addition to enlarged right atrium and ventricle

Our patient was a 35-year-old healthy African American male, who presented to the emergency department with a chief complaint of worsening cough and shortness of breath of one-week duration. He had subjective fever and was complaining of pressure-like chest pain, worse with inspiration and minimal exertion. He had been to a nearby hospital where he was tested for COVID-19 through curbside screening, and had been sent home to self-quarantine. After a few hours, due to worsening symptoms, he presented to our emergency room (ER). On presentation, his blood pressure was 102/73 mmHg, heart rate was 107 beats/minute, respiratory rate was 20 breaths/minute and he had a temperature of 36.4 Celsius. His oxygen saturation was 90% on 6L oxygen by nasal cannula. The patient was switched to 15L oxygen through a non-rebreather mask with improvement in the oxygen saturation to 99%. White blood cell (WBC) count was 9100 cells/mm3 (4000-11,000 cells/mm3), absolute lymphocyte count was 1600 cells/mm3 (1000-4800 cells/mm3), and platelet count was 370,000 cells/mm3 (150,000-450,000 cells/mm3). Creatinine was 0.93 mg/dl (0.7-1.2 mg/dl), high sensitivity troponins were 43 and 126 ng/l (3-17 ng/l), and electrolytes were within normal limits. The final results of testing for Influenza A, Influenza B, Respiratory Syncytial Virus, Pneumococcal urine antigen, Legionella urine antigen, and blood cultures were all negative. Chest x-ray showed few patchy opacities in the bilateral lung fields (Figure 1). After two hours, the patient started showing alteration in his mental status and his condition deteriorated. Arterial blood gases obtained showed severe respiratory and metabolic acidosis with a pH of 6.795 (7.35-7.45), pCO2 of 79.7 mmHg (35-45 mmHg), pO2 of 168 mmHg (80-100 mmHg) and an HCO3 of 11.8 mEq/L (22-28 mEq/L). The lactate level was 16 mmol/L (0.6-1.9 mmol/L). He was intubated and placed on ventilator support. A CT angiogram of the chest was done and showed massive pulmonary emboli in the main, segmental, and sub-segmental branches of the pulmonary arterial tree bilaterally, with findings consistent with right heart strain (Figure 2), There was also evidence of bilateral patchy ground glass opacities due to multifocal pneumonia (Figure 3). A CT scan of the head without contrast was negative for acute intracranial hemorrhage or mass effect. Sometime late, while in the ER, the patient suffered cardiopulmonary arrest, twice. Unfortunately, he passed away before time allowed for treatment with a thrombolytic agent or intravenous heparin. The COVID-19 swab result from the first evaluating hospital was reported as positive

## Discussion

Pulmonary embolus (PE) is considered the most dangerous form of venous thromboembolism (VTE). Its rate of incidence is much higher in elderly people, and is associated with high morbidity and mortality. Clinical presentation of PE is variable and can be nonspecific, making the diagnosis challenging. COVID-19 was found to increase the risks of coagulopathy. Complications reported with Severe Acute Respiratory Syndrome (SARS) caused by coronavirus in 2003-2004 included deep venous thrombosis (DVT), PE, and diffuse intravascular coagulation (DIC). Frequent pulmonary emboli in necropsy series of SARS patients were reported as well [2,3].

Excessive inflammation, hypoxia, immobilization, and DIC are the implicated factors that may predispose to both arterial and venous thromboembolism. Other suggested mechanisms include disease-specific hypercoagulable state or cytokine-mediated diffuse microvascular damage in addition to thrombocytosis [4,5].

In a study done in the Netherlands in March 2020 [6], Klok et al. evaluated the incidence of thrombotic complications in critically ill COVID-19 patients in the Intensive Care Unit (ICU). They studied 184 ICU patients with proven COVID-19 pneumonia, of whom 23 died (13%), 22 were discharged alive (12%), and 139 (76%) were still in the ICU on April 5th, 2020. All patients received at least standard doses of thromboprophylaxis. Results reported cumulative incidence of the composite outcome 31% (95% CI 20-41%), CT-Pulmonary Angiography (CT-PA) and/or ultrasonography-confirmed VTE in 27% (95% CI 17-37%), and arterial thrombotic events in 3.7% (95% CI 0-8.2%). The authors reported 25 cases with PE, three with arterial thrombotic events, two with catheter-related upper extremity thrombosis, and one with proximal leg DVT.

In another study by Poissy et al. that was done in France in April 2020 [7], 107 ICU COVID-19 patients admitted in March 2020 were compared to 196 patients admitted to the same ICU in March 2019. Despite a similar severity score on admission, the frequency of PE in COVID-19 patients was twice higher than the frequency found in the controls. Results reported that of 22 patients that developed PE, 20 were receiving prophylactic antithrombotic treatment with Unfractionated Heparin (UFH) or Low Molecular Weight Heparin (LWMH), one patient with history of DVT was receiving fluindione with international normalised ratio (INR) in the therapeutic range, and one patient was receiving therapeutic UFH due to atrial fibrillation.

It is also worth mentioning that patients with risk factors for VTE remain at higher risk, and it is unreasonable for every COVID-19 patient to undergo imaging looking for PE. Pretest probability remains very important, and well-known scoring systems as Wells’ criteria, Pulmonary Embolism Rule-out Criteria (PERC), or the Geneva scoring system should always be applied. If the patient has a reasonable probability of PE and lung parenchymal opacities are present on chest radiography, CT-PA should be obtained [8]. The International Society on Thrombosis and Hemostasis (ISTH) supports using laboratory tests such as D-dimers, prothrombin time, and platelet count to stratify patients regarding adverse outcomes and need for hospital admission [9]. According to the ISTH, all admitted patients should be on antithrombotic prophylaxis with LMWH, unless there is a contraindication.

## Conclusions

Pulmonary embolus as a complication of COVID-19 infection can be expected mostly in the elderly or in people with established risk factors, but we want to highlight that pretest probability for PE can remain high even in young, previously healthy COVID-19 patients with no risk factors for PE. We aim to emphasize this to increase awareness among healthcare professionals of the potential of PE in any patient affected with the disease. Prompt COVID-19 diagnosis and high suspicion for PE can be contributors to successful treatment, which can be life-saving.
